# Cardiovascular Actions and Therapeutic Potential of Tetramethylpyrazine (Active Component Isolated from* Rhizoma Chuanxiong*): Roles and Mechanisms

**DOI:** 10.1155/2016/2430329

**Published:** 2016-05-23

**Authors:** Ming Guo, Yue Liu, Dazhuo Shi

**Affiliations:** ^1^Centre of Cardiovascular Diseases, Xiyuan Hospital of China Academy of Chinese Medical Sciences, Beijing 100091, China; ^2^China Heart Institute of Chinese Medicine, China Academy of Chinese Medical Sciences, Beijing 100091, China

## Abstract

Tetramethylpyrazine (TMP), a pharmacologically active component isolated from the rhizome of the Chinese herb* Rhizoma Chuanxiong* (Chuanxiong), has been clinically used in China and Southeast Asian countries for the prevention and treatment of cardiovascular diseases (CVDs) for about fifty years. The pharmacological effects of TMP on the cardiovascular system have attracted great interest. Emerging experimental studies and clinical trials have demonstrated that TMP prevents atherosclerosis as well as ischemia-reperfusion injury. The cardioprotective effects of TMP are mainly related to its antioxidant, anti-inflammatory, or calcium-homeostasis effects. This review focuses on the roles and mechanisms of action of TMP in the cardiovascular system and provides a novel perspective on TMP's clinical use.

## 1. Introduction

**Figure 1:**
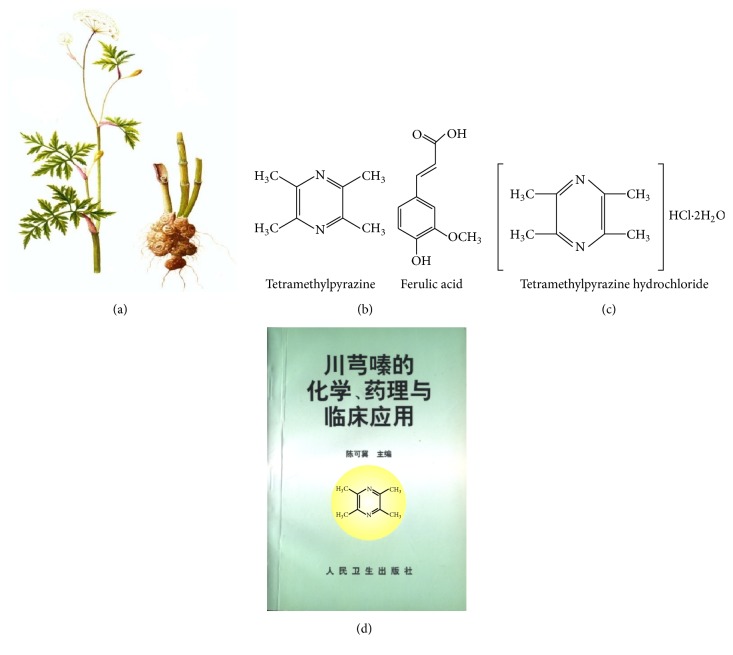
The Chuanxiong plant and the chemical structures of its major extract constituents. (a) Schematic diagram of crude* Rhizoma Chuanxiong* herb (drawn by Wang Lisheng). (b) The chemical structures of the major* Rhizoma Chuanxiong* constituents, tetramethylpyrazine and ferulic acid. (c) The chemical structure of tetramethylpyrazine hydrochloride. (d) The cover of the first academic monograph on tetramethylpyrazine published in China:* chemistry*,* pharmacology*, and* clinical application of tetramethylpyrazine,* edited by Chen Keji.

Chuanxiong, a crude herbal drug isolated from the dried root or rhizome of* Rhizoma Chuanxiong* (Figure 1(a)), has been long used in China to treat cardiovascular diseases (CVDs) including coronary heart disease, hypertension, arrhythmia, heart failure, dilated cardiomyopathy, dyslipidemia, and myocarditis [1–3]. The benefits of Chuanxiong are attributed to its vasodilating, anti-inflammatory, anticoagulant, free radical-scavenging, and microcirculatory effects [3–7]. Despite increasing amounts of research into the cardioprotective effects of Chuanxiong, the characterization of its active ingredients and the exact mechanisms underlying its therapeutic action are not fully understood. The Nobel Prize winning discoveries of Professor Tu related to artemisinin from the Chinese herb* Artemisia carvifolia* aroused global interest in Traditional Chinese Medicine (TCM). The chemical constituents and biologically active components of Chuanxiong have been widely studied since the 1930s. More than 30 compounds, having a variety of different structural types, have been isolated. Some among them fall within these three main chemical groups: alkaloids, phenolic acids, and phthalides. The alkaloid-type compounds include TMP, L-isobutyl-L-valine anhydride, and perlolyrine; the phenolic acid-type compounds include ferulic acid, coniferylferulate, and chrysophanol; and the phthalide-type compounds include Z-ligustilide, butylphthalide, 3-butylidenephthalide, senkyunolide A, and levistolide A. Among these components, TMP and ferulic acid are the more functional and structural representatives of Chuanxiong [8] (Figure 1(b)). In China, the most commonly used ligustrazine preparation is TMP hydrochloride (Figure 1(c)). The relative academic monograph about TMP,* chemistry*,* pharmacology*, and* clinical application of tetramethylpyrazine*, has now been updated, to version 2, and has had great academic influence (Figure 1(d)). Emerging experimental and clinical evidence has shown that TMP can prevent or slow the progression of a series of diseases, including CVDs, ischemic stroke, cancer, and diabetes [9–12]. In China, TMP has been used either alone or in a compound prescription for the prevention and treatment of CVDs for nearly 30 years. Herein, we provide a review of the recent experimental studies and clinical trials on the cardiac effects of TMP and its potential mechanisms of action.

## 2. TMP

### 2.1. Pharmacokinetic Properties of TMP

The pharmacokinetic properties of TMP have been widely investigated in recent years. TMP is moderately bound to plasma proteins (64.64%) [13]. It is absorbed slowly in the digestive tract. Previous studies [14] showed that the content of TMP is higher in stomach tissue than in intestinal tissue after oral administration in rats, mice, and dogs; this finding may be related to the hydrochloride and phosphate preparations in which TMP is commonly found. The pharmacokinetic properties of TMP are summarized in Table 1.

Cytochrome P450 (CYP) enzymes metabolize drugs by acting as the primary enzymes for redox reactions.* In vitro*, TMP undergoes phase I metabolism by the action of CYP1A2, 2C9, and 3A4 (CYP isoforms) in mice and human liver microsomes [18]. Using rat liver microsomes, Kuang et al. [19] reported that CYP3A plays a major role in the TMP metabolism in rats, whereas CYP2B had no effect. Treatment of LS174 T cells with TMP results in an increase in CYP3A4 mRNA owing to pregnane X receptor activation [20]. This indicates that TMP can change the pharmacokinetics of other drugs by altering their metabolism, hence modifying their activities and toxicities.

### 2.2. Drug Interactions

TMP hydrochloride or phosphate injections are TMP formulations that have been extensively used in clinics in China for nearly 30 years. Anaphylactic shock due to contamination during the production process is a rare but fatal side effect [21]. Chan et al. [22] demonstrated the synergism between TMP and nitric oxide donors leading to increased relaxation in isolated rat aortas. This interaction was associated with an increase in sodium nitroprusside (SNP), inducing relaxation mainly by Ca^2+^ sensitization. TMP should be used prudently in combination with other drugs. Since most studies are experimental, large-scale clinical trials are required to assess the efficacy of TMP in the treatment of cardiovascular disease.

## 3. Cardioprotective Effects of TMP

### 3.1. Effect on the Vasomotor Function

Endothelin (ET) and nitric oxide (NO) are the main endothelial active substances that regulate vasomotor effects. ET-1, a 21 amino acid polypeptide derived from vascular endothelial cells and smooth muscle cells, is the primary antecedent in cardiovascular disease [23–25]. Bi et al. demonstrated that TMP inhibits strain-induced ET-1 secretion, which was partially due to a reduction in reactive oxygen species (ROS) and in the phosphorylation of extracellular signal-regulated kinases (ERK) 1/2. Consequently, the activity of activator protein-1 (AP-1) reduced. The expression of the ET-1 gene also reduced, resulting in vasodilatation [26]. Lee et al. reported that TMP inhibits angiotensin II- (AngII-) induced ROS generation, ERK phosphorylation, and ET-1 gene expression in vascular endothelial cells [27]. Endothelial NOS synthesizes NO, which diffuses into adjacent vascular smooth muscle cells, initiating the conversion of GTP to cGMP and causing vasodilatation [28].* In vivo*, TMP increased the concentration of intraendothelial Ca^2+^, which may trigger the expression of eNOS, increasing the levels of NO and ameliorating the vasospasms [29]. These observations support that TMP has effects on vasomotor function.

### 3.2. Effect on Platelet Profile

There are abundant membrane glycoproteins on the platelet surface, which mediate adhesion, activation, and aggregation of platelets, resulting in thromboses. Studies monitoring intracellular ionized calcium have demonstrated that TMP inhibits calcium mobilization from both the extracellular medium and intracellular stores, creating a potent antiplatelet effect [30, 31]. Sheu et al. investigated the possible mechanism responsible for TMP antiplatelet activity using human platelets. The results indicated that the antiplatelet activity of TMP may involve two pathways: at a lower centration (0.5 mM), TMP inhibits phosphoinositide breakdown and thromboxane A_2_ formation, and at a higher concentration (1.0 mM), it inhibits platelet aggregation by binding to the glycoprotein IIb/IIIa complex [32]. Another study demonstrated that TMP (50–200 *μ*M) significantly increases the production of nitrate and cyclic GMP in human platelets within a 15 min incubation period. Moreover, TMP inhibits intracellular Ca^2+^ mobilization in human platelets stimulated by collagen in a concentration-dependent manner [33]. Li et al. [34] reported that under high shear rates TMP exerts antiplatelet effects by inhibiting the vWF-mediated process of platelet thrombus formation.

### 3.3. Effect on Oxidation and Proliferation

Oxidative stress and ROS are key pathogenic factors involved in endothelial cell injury and cardiovascular disease [35]. Li et al. assessed the protective effect of TMP on H_2_O_2_-induced human umbilical vein endothelial cells (HUVECs). The results suggested that TMP reduced intracellular nitric oxide, malondialdehyde, and nitric oxide synthase levels owing to its antioxidant properties [36, 37]. Wong et al. demonstrated that TMP inhibits AngII-induced proliferation and ET-1 activity, partially by interfering with the ERK pathway via attenuation of AngII and consequent reduction of NAD(P)H oxidase-induced ROS generation [38]. Kang et al. reported that TMP can protect endothelial cells from high glucose levels, which cause damage to the cells via ROS production, downregulation of Akt (protein kinase B)/eNOS phosphorylation, and reduction of NO generation, and is partially attributed to uncoupling protein 2 (UCP2) mRNA/protein expression [39].

Vascular smooth muscle cell (VSMC) proliferation plays a pivotal role in the occurrence and development of atherosclerosis and vascular restenosis. Di et al. [40] demonstrated that the proliferation of rat VSMCs induced by platelet-derived growth factor (PDGF) can be significantly inhibited by TMP in a dose-dependent manner. Meanwhile, a remarkable decrease of AP-1 and PCNA (proliferation cell nuclear antigen) expression was seen in the TMP group. Zheng et al. [41] reported that the VSMC proliferation induced by AngII can be inhibited by TMP in a dose-dependent manner and that the responsible mechanism may be correlated with the inhibition of calcineurin (CaN) activities and the reduction in e-fos and PCNA expression. Another study showed that TMP inhibited VEGF-induced HUVEC migration and tube formation and also suppressed VEGF-induced rat aortic ring sprouting in a dose-dependent manner [42]. Ma et al. [43] compared the effect of a TMP-eluting stent (TES) with that of a control bare metal stent in a porcine coronary stent restenosis model; the results revealed that TES inhibited neointimal hyperplasia and reduced in-stent restenosis, which provided support for its clinical application.

### 3.4. Effect on Atherosclerosis

The effects of TMP on the critical components of atherogenesis have recently been intensively investigated. Ma et al. [44] reported that TMP could inhibit atherosclerosis in Apo-E knockout mice fed a high-fat diet by activating transcription factor nuclear factor E2-related factor 2 (Ncf-2), which is an important antioxidative stress factor. This was also confirmed in another study [45]. Furthermore, Jiang et al. [46] demonstrated that TMP suppresses the development of atherosclerosis and hepatic lipid accumulation via the alleviation of oxidative stress and dyslipidemia. Wang et al. [47] showed that TMP decreases monocyte chemoattractant protein-1 (MCP-1) and intercellular adhesion molecule-1 (ICAM-1) levels in the plasma and inhibits LOX-1 expression in rabbit aortas. Likewise, in their* in vitro* study, they revealed that TMP suppresses the ox-LDL-induced activation of p-ERK, p-p38, and p-JNK MAPK.* In vitro*, HUVECs were stimulated with TNF-*α*, and two important indicators of autoimmunity, ICAM-1 and heat shock protein 60 (HSP60), were evaluated to determine the influence of TMP on HUVECS. The results suggested that TMP protects the endothelium via inhibition of immunological reactions, preventing atherosclerosis [48].

### 3.5. Effect on Calcium Channels

Intracellular Ca^2+^ homeostasis is necessary to maintain cell function. Ischemia/reperfusion can lead to Ca^2+^ influx and disorders in the mechanisms responsible for Ca^2+^ gradients, which could result in myocardial Ca^2+^ overload [49]. It has been reported that TMP not only blocks the entry of extracellular calcium through calcium channels but also inhibits the release of intracellular calcium stores in vascular smooth muscle cells [50]. Tsai et al. investigated the effects of TMP on calcium influx in cultured vascular smooth muscle (A7r5) cells using the fura-2 indicator. The results indicated that TMP opens potassium channels lowering calcium influx into the cultured aortic smooth muscle cells [51]. Other studies have consistently shown that TMP inhibits I_Ca-L_ in a concentration-dependent manner in both rat [52] and rabbit [53] ventricular myocytes. These observations suggest that TMP has a role in calcium-homeostasis and further supports its clinical use in patients with coronary heart disease.

### 3.6. Effect on the Inflammatory Responses

The relationship between inflammatory responses and coronary heart disease has been well established [54]. Inflammatory injury is activated in ischemic periods and aggravated upon reperfusion. Li and colleagues found that TMP inhibits LPS-induced IL-8 production in HUVECs at both the mRNA and protein level. Furthermore, they found that this is mediated by the NF-*κ*B-dependent pathway. TMP also affected the ERK and p38 MARK pathways [55]. Lv et al. demonstrated that TMP increases SOD activity and eNOS mRNA and protein expression and decreases MDA content, MPO activity, and IL-1*β* levels in the myocardial tissue [4]. Zhou et al. [56] reported that TMP promotes the activity of SOD and GSH-Px and decreases that of MDA, LDH, creatinine kinase, tumor necrosis factors-*α*, and IL-6. Wang et al. [57] demonstrated that TMP can significantly decrease the gene expression of ICAM-1 and protein expression of cyclooxygenases-2 (Cox-2), by acting on the MAPK and NF-*κ*B signaling pathways.

### 3.7. Effect on Ischemia-Reperfusion Injury

Previous studies reported that platelet aggregation, inflammatory response, microembolization, and cell death contribute to myocardial ischemia-reperfusion injury [58]. A low dose of TMP (20 mg/10 mg·kg^−1^·d^−1^) can reduce myocardial pathology injury, increase the Ca^2+^-ATPase activity of myocardial mitochondria, improve cardiac function, and antagonize calcium overload in rats [59]. Lv et al. [1] reported that TMP has antiapoptotic and cardioprotective effects against myocardial ischemia/reperfusion injury, which is mediated by the PI3K/Akt pathway. In addition, TMP could promote the phosphorylation of eNOS to increase NO production. Liu et al. [60] demonstrated that TMP can reduce the scope of the myocardial infarction induced by long-term ischemia, all the while decreasing hemorheological indices of myocardial ischemia in rats and protecting acute ischemic myocardium and ischemia-reperfusion injured myocardium. Another study [61] showed that TMP reduces the size of infarcts resulting from ischemia/reperfusion injury* in vivo*, which might be associated with its antioxidant activity via the induction of heme oxygenase-1 (HO-1) and with its capacity for neutrophil inhibition.

## 4. TMP and Cardiovascular Diseases

### 4.1. TMP and Coronary Heart Disease

Wang et al. [62] observed the effects of Danshen Chuanxiong injections (DCI) on myocardial damage in patients with unstable angina (UA) undergoing percutaneous coronary intervention (PCI). The results showed that the DCI group had decreased P-selectin-positive expression and lower levels of CK-MB and TnI than those in the control group. Sun [63] recruited 96 UA patients, who were randomly divided into two groups. Both were treated with conventional treatment, while one group also received ligustrazine hydrochloride injections (6 mL–10 mL + 250 mL 5% glucose injection). The results showed that this additional treatment improved the curative effects on the angina symptoms and related markers of electrocardiogram. Huang [64] also observed the clinical effect and safety of Danshen Chuanxiong injection in the treatment of patients with UA. The total effective rate of improved electrocardiograph of treatment group was significantly higher than that of the control group.

There are few randomized controlled trials addressing the effects of TMP on UA patients. Song et al. [65] compared TMP alone to a TMP and L-arginine combination in rats with acute myocardial infarction (AMI). The results showed that serum CK and TnI levels, as well as MPO concentrations, in the myocardial tissue were significantly lower than those in the control group. These inhibitory effects were closely related to the expression of adhesive factors and the decrease of leucocyte infiltration. As reported by Meng et al. [66], TMP could affect VEGF expression in rat myocardial infarction, promote endothelial cell proliferation, and augment the quantity of vessels on chick embryo chorioallantoic membranes (CAM), suggesting that Chuanxiong has angiogenic effects. Salvia TMP injections were used to investigate the effects of TMP on inflammatory cytokines and vascular endothelial function related factors in patients with AMI. The salvia TMP injections decreased hs-CRP and WBC, IL-6 levels, while elevating NO levels. Hence, it might improve vascular endothelial function in AMI patients [67]. Chen et al. [68] explored the effect of TMP on platelet activation and vascular endothelial function after PCI in patients with ACS. The results showed that TMP decreases CD62p, CD63, GPIIb/IIIa, vWF, and ET-1 levels.

### 4.2. TMP and Hypertension

TMP significantly lowered caudal artery systolic blood pressure in spontaneously hypertensive rats. This may be attributed to TMP's antioxidant effect [69]. Zheng et al. [70] observed the effect of TMP on the hemorheology in pressure overload-induced hypertensive rats. The results suggested that pressure overload could induce hypertension and increase blood viscosity, platelet aggregation, hematocrit, and rigidity of erythrocyte, changes which could be prevented by high concentrations of TMP. TMP was also used for treatment of preeclampsia. Chen et al. [71] reported that TMP could improve the endocrinological function of vascular endothelial cells in mothers; however, the change in the mean arterial pressure did not change with TMP treatment. Wang and Zhao [72] also found that TMP injection inhibits fibrinolysis and promotes microcirculation in pregnancy-induced hypertension patients. Some studies clearly demonstrated that TMP could inhibit VSM Ca^2+^ influx in hypertensive rats, while oral administration activated VSM Ca^2+^ influx in normal rats and inhibited VSM Ca^2+^ influx in hypertensive rats [73]. Hence, more clinical trials and experimental studies are needed to address the effect of TMP on hypertension.

### 4.3. TMP and Arrhythmia

Pretreatment with TMP (12 mg/kg/day) remarkably reduced the incidence of ischemia-induced fibrillation (VF) and ventricular tachycardia (VT) in control hearts from 100% and 50% to 41% and 0%, respectively. TMP also produced a slight, but prominent increase of 6-keto-PGF1-alpha and a decrease in TXB2 production during aerobic perfusion [74]. Zuo et al. [75] investigated the effects of TMP (20 mg/L) on reperfusion arrhythmias in the isolated Langendorff hearts of rats. The results showed that TMP could reduce the incidence and duration of reperfusion arrhythmias. TMP (20 mg/kg and 40 mg/kg) was injected into conscious rats for 5 min, followed by 30 min ischemia and 120 min reperfusion; the incidence of VT and VF and the mean duration of VT and VF were decreased [76]. Furthermore, a study has reported that TMP (40 mg/kg) can antagonize arrhythmias caused by ouabain and Cacl_2_ [77].

### 4.4. TMP and Heart Failure

Experimental investigations [78] showed that TMP reduces the degree of fibrosis in the atrial tissue of dogs with congestive heart failure, which may be related to the mechanism that decreases AF frequency and duration. Using a diastolic heart failure model, Zhang et al. [79] studied the effects of different doses of TMP on myocardial cell calcium overload and injury. The results suggested that a low dosage of TMP could reduce myocardial pathology injury, increase Ca^2+^-ATPase activity in the myocardial mitochondria, improve cardiac function and Ca^2+^ level in the cardiocytes, and antagonize calcium overload in rats with decompensated heart failure. Chen and Zhou [80] reported that, in chronic congestive heart failure patients, treatment with an injection of TMP in addition to an* Astragalus membranaceus* extract increased patients' cardiac myocardial contraction force and output, significantly improving the symptoms of heart failure. TMP can antagonize vasoconstriction due to its effects on AngII and other vasopressin factors, ultimately blocking the pathogenesis of the heart failure.

### 4.5. TMP and Dilated Cardiomyopathy

Dilated cardiomyopathy (DCM) is a primary heart muscle disease characterized by dilation of the heart chambers and markedly reduced heart contractions [81]. Zhao et al. investigated the effect of TMP on the progression of DCM in cTnT^R141W^ transgenic mice; the results showed that TMP remarkably prevents the cardiac dilatation and dysfunction which develops in DCM and decreases mortality by 54%. TMP decreased heart weight/boy weight ratios and the expression of hypertrophic markers BNP and ACTA1, as well as reducing interstitial collagen deposition and the expression of profibrotic markers Col1a1 and Col3a1. TMP attenuated the ultrastructural disruption caused by cTnT^R141W^ expression and decreased the expression of structural proteins myotilin and E-cadherin, which are upregulated in cTnT^R141W^ hearts. Lu et al. [82] demonstrated that treatment with Rb1 and TMP had synergistic effects on the amelioration of chamber dilation, interstitial fibrosis, contractile dysfunction, and ultrastructural degeneration in cTnT^R141W^ mice, which was probably due to the inhibition of both HB-EGF and the Ca2^+^/CaM/CaMKII pathway. Another study showed that TMP inhibits AngII-induced cardiomyocyte hypertrophy and TNF-*α* production via suppression of the NF-*κ*B pathway. This could provide new insight into the mechanisms underlying the protective effects of TMP in heart diseases [83].

### 4.6. TMP and Dyslipidemia

Fu et al. [84] investigated the therapeutic effects and mechanisms of TMP on streptozotocin-induced-nephropathy in type 2 diabetic rats. The results showed that TMP (80 and 160 mg/kg) can significantly decrease the levels of total cholesterol (TC), triglyceride (TG), and low-density lipoprotein (LDL-c) in the serum and increase the levels of high-density lipoprotein (HDL-c) in the blood. Jiang et al. [46] also demonstrated that TMP can decrease TC, TG, and LDL-c levels, while increasing HDL-c levels in the plasma of rats. Furthermore, histological examinations revealed that TMP suppresses atherosclerotic plaque progression in the thoracic aortas and lipid accumulation in the livers of rats. Yang et al. [85] investigated the effects of TMP on lipid and apoprotein in nephrotic syndrome in children; the results suggested TMP (8–10 mg/kg) decreases TC, TG, LDL-C, VLDL-C, and apoB levels, all the while increasing HDL-C and apoA1 levels.

### 4.7. TMP and Viral Myocarditis

To explore the effect of TMP on myocarditis, Jiang et al. monitored cardiomyocyte apoptosis in mice infected with Coxsackie virus B3 (CVB3) and found that the TMP treatment group had reduced positive staining indexes of Fas/FasL in their cardiomyocytes [86]. Qian and colleagues studied the protective effects of TMP on rat myocardial cells infected with CVB3 and its signal transduction mechanism; they found that TMP could increase the contraction rate of myocardial cells and elevate LDH activity and NF-*κ*B expression [87]. Ni [88] observed the clinical effect of Shenmai in combination with TMP injections on children with acute viral myocarditis (VMC). They found the conventional treatment and Shenmai and TMP injections could ameliorate the clinical symptoms and ECGs. Another study discussed the therapeutic effect of levocarnitine (50–100 mg/kg) plus TMP injection (40 mg/d) on children with VMC. The results showed that the combination of levocarnitine and TMP could ameliorate clinical signs and symptoms, and the ECG and echocardiography gradually returned to the normal [89].

## 5. Conclusions and Perspectives

As reported above, there is a solid amount of clinical and experimental evidence suggesting that TMP holds great promise for the treatment of CVDs. Ongoing research on TMP may broaden its potential clinical uses all over the world. Combination strategies play an important role in the prevention and treatment of CVDs. Based on its cardiovascular pharmacological effects, clinicians should pay close attention to the potential combination therapies amalgamating TMP with antiplatelet drugs or statins. The clinical studies reported in this review were mainly performed on small sample groups. Large, randomized, controlled, and double blind clinical trials are required to fully address the potential clinical roles and the mechanisms of TMP in CVDs. On the other hand, TMP is rapidly metabolized and has a short half-life and low bioavailability. The development of a series of TMP derivatives is a hot topic, attracting many scholars all over the world. More effective TMP preparations will undoubtedly be developed for the future prevention and treatment of CVDs.

## Figures and Tables

**Table 1:** Pharmacokinetic properties of TMP.

Species	Dose	PK parameters	References
SD rats	10 mg/kg (i.v.)	*t*_1/2,*α*_ 14.4 ± 1.2 min	[15]
		*t*_1/2,*β*_ 28.7 ± 4.1 min	
		AUC 82.1 ± 14.9 *µ*g min/mL	
		Cl 0.15 ± 0.044 (L/kg/min)	
	10 mg/kg (i.n.)	*t*_1/2_ 49.2 ± 20.5 min	[16]
		*C*_max_ 9.4 ± 4.1 min	
		*T*_max_ 9.2 ± 2.0 min	
		AUC_0–*∞*_ 742.1 ± 157.1 min *μ*g/mL	
		Cl 0.014 ± 0.003 L/min/kg	
	20.26 mg/kg (p.o.)	*T*_max_ 0.9 ± 0.22 h	[17]
		*t*_1/2_ 8.04 ± 2.92 h	
		*C*_max_ 8.84 ± 2.8 ng/mL	
		Cl 125 ± 34 mL/kg/min	

## References

[1] Lv L., Jiang S.-S., Xu J., Gong J.-B., Cheng Y. (2012). Protective effect of ligustrazine against myocardial ischaemia reperfusion in rats: the role of endothelial nitric oxide synthase. *Clinical and Experimental Pharmacology and Physiology*.

[2] Huang W.-D., Yang Y.-F., Chen J.-W., Zhu B.-H. (2013). Synergism of tanshinol and tetramethylpyrazine on cardiovascular system in rats. *Chinese Pharmacological Bulletin*.

[3] Kwan C. Y., Daniel E. E., Chen M. C. (1990). Inhibition of vasoconstriction by tetramethylpyrazine: does it act by blocking the voltage-dependent Ca channel?. *Journal of Cardiovascular Pharmacology*.

[4] Lv L., Meng Q.-X., Jiang S.-S. (2012). The effects of ligustrazine on attenuating inflammatory reaction of myocardial ischemia reperfusion injury in rats and its mechanism. *Chinese Journal of Arteriosclerosis*.

[5] Guo L., Wang A. H., Sun Y. L., Xu C. G. (2012). Evaluation of antioxidant and immunity function of tetramethylpyrazine phosphate tablets in Vivo. *Molecules*.

[6] Li M., Handa S., Ikeda Y., Goto S. Y. (2001). Specific inhibiting characteristics of tetramethylpyrazine, one of the active ingredients of the Chinese herbal medicine ‘Chuanxiong,’ on platelet thrombus formation under high shear rates. *Thrombosis Research*.

[7] Ceylan-Isik A. F., Fliethman R. M., Wold L. E., Ren J. (2008). Herbal and traditional Chinese medicine for the treatment of cardiovascular complications in diabetes mellitus. *Current Diabetes Reviews*.

[8] Xiao Y.-Q., Li L., You X.-L., Taniguchi M., Baba K. (2002). Studies on chemical constituents of the rhizomae of Ligusticum chuanxiong. *Zhongguo Zhong Yao Za Zhi*.

[9] Zhao Z. H., Moghadasian M. H. (2008). Chemistry, natural sources, dietary intake and pharmacokinetic properties of ferulic acid: a review. *Food Chemistry*.

[10] Wu B., Liu M., Liu H. (2007). Meta-analysis of traditional Chinese patent medicine for ischemic stroke. *Stroke*.

[11] Wang P. L., She G. M., Yang Y. N. (2012). Synthesis and biological evaluation of new ligustrazine derivatives as anti-tumor agents. *Molecules*.

[12] Lin L. N., Wang W. T., Xu Z. J. (1997). Clinical study on ligustrazine in treating myocardial ischemia and reperfusion injury. *Zhongguo Zhong Xi Yi Jie He Za Zhi*.

[13] Ye Y., Zhou L.-L., Yan Y.-L., Huang Q.-J. (2010). Determination of plasma protein binding rate of tetramethylpyrazine phosphate by ultrafiltration. *Journal of Chinese Medicinal Materials*.

[14] Lou Y. Q., Zhang H., Cao X., Chen M. L. (1986). The pharmacokinetics and disposition of tetramethylpyrazine phosphate in dogs and rats. *Acta Pharmaceutica Sinica*.

[15] Tsai T.-H., Liang C.-C. (2001). Pharmacokinetics of tetramethylpyrazine in rat blood and brain using microdialysis. *International Journal of Pharmaceutics*.

[16] Wang Q., Tang Z., Zhang W. (2013). Brain microdialysate, CSF and plasma pharmacokinetics of ligustrazine hydrochloride in rats after intranasal and intravenous administration. *Biopharmaceutics and Drug Disposition*.

[17] Zeng M., Zhang J., Yang Y. (2014). An automated dual-gradient liquid chromatography-MS/MS method for the simultaneous determination of ferulic acid, ligustrazine and ligustilide in rat plasma and its application to a pharmacokinetic study. *Journal of Pharmaceutical and Biomedical Analysis*.

[18] Tan Y., Zhuang X.-M., Shen G.-L., Li H., Gao Y. (2014). Investigation of metabolic kinetics and reaction phenotyping of ligustrazin by using liver microsomes and recombinant human enzymes. *Yao Xue Xue Bao*.

[19] Kuang X.-D., Li X.-H., Xiong Y.-Q. (2006). Study on metabolism of tetramethylpyrazine in system of rat liver microsomes. *Zhongguo Zhong Yao Za Zhi*.

[20] Zhang Y.-W., Bao M.-H., Wang G., Qu Q., Zhou H.-H. (2014). Induction of human CYP3A4 by huperzine A, ligustrazine and oridonin through pregnane X receptor-mediated pathways. *Pharmazie*.

[21] Xiong X. M., Geng J. Q. (2010). One case of Ligustrazine phosphate injection induing anaphylactic shock. *Chinese Journal of Pharmacoepidemiology*.

[22] Chan S. S.-K., Jones R. L., Lin G. (2009). Synergistic interaction between the Ligusticum chuanxiong constituent butylidenephthalide and the nitric oxide donor sodium nitroprusside in relaxing rat isolated aorta. *Journal of Ethnopharmacology*.

[23] Schiffrin E. L. (2001). Role of endothelin-1 in hypertension and vascular disease. *American Journal of Hypertension*.

[24] Schiffrin E. L., Touyz R. M. (1998). Vascular biology of endothelin. *Journal of Cardiovascular Pharmacology*.

[25] Mundhenke M., Schwartzkopff B., Köstering M., Deska U., Klein R. M., Strauer B. E. (1999). Endogenous plasma endothelin concentrations and coronary circulation in patients with mild dilated cardiomyopathy. *Heart*.

[26] Bi W.-F., Yang H.-Y., Liu J.-C. (2005). Inhibition of cyclic strain-induced endothelin-1 secretion by tetramethylpyrazine. *Clinical and Experimental Pharmacology and Physiology*.

[27] Lee W.-S., Yang H.-Y., Kao P.-F. (2005). Tetramethylpyrazine downregulates angiotensin II-induced endothelin-1 gene expression in vascular endothelial cells. *Clinical and Experimental Pharmacology and Physiology*.

[28] Murad F. (2006). Shattuck Lecture. Nitric oxide and cyclic GMP in cell signaling and drug development. *The New England Journal of Medicine*.

[29] Shao Z., Li J., Zhao Z., Gao C., Sun Z., Liu X. (2010). Effects of tetramethylpyrazine on nitric oxide/cGMP signaling after cerebral vasospasm in rabbits. *Brain Research*.

[30] Liu S.-Y., Sylvester D. M. (1990). Antithrombotic/antiplatelet activity of tetramethylpyrazine. *Thrombosis Research*.

[31] Zhou X. B., Salganicoff L., Sevy R. (1985). The pharmacological effect of ligustrazine on human platelets. *Yao Xue Xue Bao*.

[32] Sheu J.-R., Kan Y.-C., Hung W.-C., Ko W.-C., Yen M.-H. (1997). Mechanisms involved in the antiplatelet activity of tetramethylpyrazine in human platelets. *Thrombosis Research*.

[33] Sheu J.-R., Kan Y.-C., Hung W.-C., Lin C.-H., Yen M.-H. (2000). The antiplatelet activity of tetramethylpyrazine is mediated through activation of NO synthase. *Life Sciences*.

[34] Li M., Handa S., Ikeda Y., Goto S. (2001). Specific inhibiting characteristics of tetramethylpyrazine, one of the active ingredients of the Chinese herbal medicine ‘Chuanxiong,’ on platelet thrombus formation under high shear rates. *Thrombosis Research*.

[35] Heitzer T., Schlinzig T., Krohn K., Meinertz T., Münzel T. (2001). Endothelial dysfunction, oxidative stress, and risk of cardiovascular events in patients with coronary artery disease. *Circulation*.

[36] Li W.-M., Liu H.-T., Li X.-Y. (2010). The effect of tetramethylpyrazine on hydrogen peroxide-induced oxidative damage in human umbilical vein endothelial cells. *Basic and Clinical Pharmacology and Toxicology*.

[37] Ou Y., Guo X.-L., Zhai L., Liu X.-Y., Cheng Y.-N. (2010). TMPDP, a tetramethylpyrazine derivative, protects vascular endothelial cells from oxidation damage by hydrogen peroxide. *Pharmazie*.

[38] Wong K.-L., Wu K.-C., Wu R. S.-C., Chou Y.-H., Cheng T.-H., Hong H.-J. (2007). Tetramethylpyrazine inhibits angiotensin II-increased NAD(P)H oxidase activity and subsequent proliferation in rat aortic smooth muscle cells. *The American Journal of Chinese Medicine*.

[39] Kang Y., Hu M., Zhu Y., Gao X., Wang M.-W. (2009). Antioxidative effect of the herbal remedy Qin Huo Yi Hao and its active component tetramethylpyrazine on high glucose-treated endothelial cells. *Life Sciences*.

[40] Di K.-P. D., Jia X.-C., Qi R. (2010). Study on the molecular Mechanism of Ligustrazine inhibiting proliferation of vascular smooth muscle cells. *Chinese Journal of Basic Medicine in Traditional Chinese Medicine*.

[41] Zheng H. H., Luo D. S., Li Y. H. (2006). Interference effects of tatramethylpyrazine on the proliferation of vascular smooth muscle cells induced by angiotensin II. *Medical Journal of Chinese People's Liberation Army*.

[42] Chen L., Lu Y., Wu J.-M. (2009). Ligustrazine inhibits B16F10 melanoma metastasis and suppresses angiogenesis induced by Vascular Endothelial Growth Factor. *Biochemical and Biophysical Research Communications*.

[43] Ma G., Ding S., Feng Y., Shen C., Chen L., Chen Z. (2007). Tetramethylpyrazine-eluting stents prevented in-stent restenosis in a porcine model. *Journal of Cardiovascular Pharmacology*.

[44] Ma H., Ren W.-Y., Yuan Y., Shen J.-P., Hu Y. (2015). The inhibitory effects of tetramethylpyrazine (TMP) on atherosclerosis in Apo-E knock-out and high-fat-diet fed mice by activating nuclear factor E_2_-related factor-2 (Nrf-2). *Fudan University Journal of Medical Sciences*.

[45] Feng L., Ke N., Cheng F. (2011). The protective mechanism of ligustrazine against renal ischemia/reperfusion injury. *Journal of Surgical Research*.

[46] Jiang F., Qian J., Chen S., Zhang W., Liu C. (2011). Ligustrazine improves atherosclerosis in rat via attenuation of oxidative stress. *Pharmaceutical Biology*.

[47] Wang G.-F., Shi C.-G., Sun M.-Z. (2013). Tetramethylpyrazine attenuates atherosclerosis development and protects endothelial cells from ox-LDL. *Cardiovascular Drugs and Therapy*.

[48] Wu H.-J., Hao J., Wang S.-Q., Jin B.-L., Chen X.-B. (2012). Protective effects of ligustrazine on TNF-*α*-induced endothelial dysfunction. *European Journal of Pharmacology*.

[49] Ruiz-Meana M., García-Dorado D. (2009). Pathophysiology of ischemia-reperfusion injury: new therapeutic options for acute myocardial infarction. *Revista Espanola de Cardiologia*.

[50] Pang P. K. T., Shan J. J., Chiu K. W. (1996). Tetramethylpyrazine, a calcium antagonist. *Planta Medica*.

[51] Tsai C.-C., Lai T.-Y., Huang W.-C. (2003). Tetramethylpyrazine as potassium channel opener to lower calcium influx into cultured aortic smooth muscle cells. *Planta Medica*.

[52] Zou L. Y., Hao X. M., Zhang G. Q., Zhang M., Guo J., Liu T. (2001). Effect of tetramethyl pyrazine on L-type calcium channel in rat ventricular myocytes. *Canadian Journal of Physiology and Pharmacology*.

[53] Ren Z., Ma J., Zhang P. (2012). The effect of ligustrazine on L-type calcium current, calcium transient and contractility in rabbit ventricular myocytes. *Journal of Ethnopharmacology*.

[54] Frangogiannis N. G., Smith C. W., Entman M. L. (2002). The inflammatory response in myocardial infarction. *Cardiovascular Research*.

[55] Li X.-Y., He J.-L., Liu H.-T., Li W.-M., Yu C. (2009). Tetramethylpyrazine suppresses interleukin-8 expression in LPS-stimulated human umbilical vein endothelial cell by blocking ERK, p38 and nulear factor-*κ*B signaling pathways. *Journal of Ethnopharmacology*.

[56] Zhou Y., Hu C.-P., Deng P.-Y., Deng H.-W., Li Y.-J. (2004). The protective effects of ligustrazine on ischemia-reperfusion and DPPH free radical-induced myocardial injury in isolated rat hearts. *Planta Medica*.

[57] Wang F., Lu F., Zhao Z., Yang C.-H. (2012). Effects of Ligustrazine on inflammation induced by oxidized low density lipoprotein in endothelial cells. *Chinese Journal of Hypertension*.

[58] Turer A. T., Hill J. A. (2010). Pathogenesis of myocardial ischemia-reperfusion injury and rationale for therapy. *The American Journal of Cardiology*.

[59] Zhang X., Liu W., Zhou J., Fan C. (2009). Studies on protection and mechanism of tetramethylpyrazine on myocardial injury of rats with DHF. *China Journal of Chinese Materia Medica*.

[60] Liu X., Liu H., Zeng Z., Zhou W., Liu J., He Z. (2011). Pharmacokinetics of ligustrazine ethosome patch in rats and anti-myocardial ischemia and anti-ischemic reperfusion injury effect. *International Journal of Nanomedicine*.

[61] Chen S.-Y., Hsiao G., Hwang H.-R., Cheng P.-Y., Lee Y.-M. (2006). Tetramethylpyrazine induces heme oxygenase-1 expression and attenuates myocardial ischemia/reperfusion injury in rats. *Journal of Biomedical Science*.

[62] Wang R., Han Q.-H., Jia Y.-P. (2011). Effect of danshen chuanxiongqin injection on the myocardial damage of unstable angina patients undergoing percutaneous coronary intervention. *Chinese Journal of Integrated Traditional and Western Medicine*.

[63] Sun D. N. (2007). Analysis of therapeutic effect of Chuanxiongqi glucose injection in curing unstable angina. *Journal of Practical Medical Techniques*.

[64] Huang F. M. (2008). Effect observation on danshen chuanxiongqin injection on treating patients with unstable angina pectoris. *Journal of Bethune Military Medical College*.

[65] Song Z.-J., Chen Q., Li B.-L. (2004). Protective effect of tetramethylpyrazine and L-arginine on rats with acute myocardial infarction. *Zhongguo Zhong Xi Yi Jie He Za Zhi*.

[66] Meng H., Guo J., Sun J.-Y. (2008). Angiogenic effects of the extracts from Chinese herbs: angelica and ChuanXiong. *American Journal of Chinese Medicine*.

[67] Xia K. H., Yin Z. J., Jin Y. M. (2015). Effect of Salvia Tetramethylpyrazine injection on circulation inflammatory cytokines and vascular endothelial function in patients with acute myocardial infarction. *Chinese Journal of Critical Care Medicine*.

[68] Chen Z.-Q., Hong L., Wang H. (2007). Effect of tetramethylpyrazine on platelet activation and vascular endothelial function in patients with acute coronary syndrome undergoing percutaneous coronary intervention. *Zhongguo Zhong Xi Yi Jie He Za Zhi*.

[69] Lai L. L., Rao L. Q. (2013). Effect of Tetramethylpyrazine on spontaneously hypertensive rats (SHR). *Asia-Pacific Traditional Medicine*.

[70] Zheng H. Z., Chen X. Y., Liang H. Y. (2006). Effects of tetramethylpyrazine on the hemorheology in pressure overload-induced hypertensive rats. *Chinese Journal of Hemorheology*.

[71] Chen D. J., Zhao Y., Zuo R. Z. (2003). Comparative study on effect of Ligustrazin and Magnesium sulfate on patients with pregnancy induced hypertension syndrome. *Chinese Journal of Integrated Traditional and Western Medicine*.

[72] Wang X. F., Zhao M. Q. (2003). Ligustrazine and Salvia miltiorrhiza injection solution in complementary therapy of pregnancy-induced hypertension: clinical analysis of 60 cases. *Journal of First Military Medical University*.

[73] Xu S. B., Wen Y. Y., Qin M. Q. (1989). Effect of Ligustrazine on dynamica of Ca^2+^ influx in arteries from normotensive and hypertensive rats. *Chinese Journal of Applied Physiology*.

[74] Feng J., Wu G., Tang S. (1999). The effects of tetramethylpyrazine on the incidence of arrhythmias and the release of PGI2 and TXA2 in the ischemic rat heart. *Planta Medica*.

[75] Zuo B. H., Zhou Z. Y., Zhao G. S., Yang H. J. (1996). Action of *Salvia miltiorrhiza* and tetramethylpyrazine on reperfusion arrhythmias in the isolated perfused rat heart. *Pharmacology and Clinics of Chinese Materia Medica*.

[76] Liang R. X., Liao F. L., Han D. (2002). Ligustrazini preconditioning protection on myocardial ischemia and reperfussion injury in the conscious rat. *Chinese Journal of Experimental Traditional Medical Formulae*.

[77] Chen Z. F., Liao S. H., Luo Q. (1988). Effect of Ligustrazine on Ouabain and arrhythmia induced by calcium chloride. *Chinese Pharmacological Bulletin*.

[78] Lin Y.-Z., Xu C.-X., Deng Y.-L., Chen L., Huang H., Du J. (2006). Effects of tetramethylpryrazine on fibrosis of atrial tissue and atrial fibrillation in a canine model of congestive heart failure induced by ventricular tachypacing. *Journal of Chinese Integrative Medicine*.

[79] Zhang X., Liu W., Zhou J., Fan C. (2009). Studies on protection and mechanism of tetramethylpyrazine on myocardial injury of rats with DHF. *Zhongguo Zhongyao Zazhi*.

[80] Chen Z., Zhou F. X. (2011). Effect of Astragalus injection and Ligustrazine injection in treatment of chronic congestive heart failure. *Journal of Medical Forum*.

[81] Richardson P., Mckenna R. W., Bristow M. (1996). Report of the 1995 world health organization/international society and federation of cardiology task force on the definition and classification of cardiomyopathies. *Circulation*.

[82] Lu D., Shao H.-T., Ge W.-P. (2012). Ginsenoside-Rb1 and tetramethylpyrazine phosphate act synergistically to prevent dilated cardiomyopathy in cTnTR141W transgenic mice. *Journal of Cardiovascular Pharmacology*.

[83] Yu L., She T., Li M., Shi C., Han L., Cheng M. (2013). Tetramethylpyrazine inhibits angiotensin II-induced cardiomyocyte hypertrophy and tumor necrosis factor-*α* secretion through an NF-*κ*B-dependent mechanism. *International Journal of Molecular Medicine*.

[84] Fu Y.-J., Zhou Y., Pan J.-Q., Zhang X.-M., Lü J.-H. (2012). The rapeutic effects and mechanisms of tetramethylpyrazine on streptozocin-induced-nephropathy in type 2 diabetic rats. *Chinese Pharmaceutical Journal*.

[85] Yang Q. Z., Lv X. Y., Yu A. J. (2002). Clinical study on the changes of serum lipids and apolipoprotein in patients with nephrotic syndrome. *Chinese Journal of Contemporary Pediatrics*.

[86] Jiang H., Chen X. X., Wang J. Y. (2014). Effect of Ligustrazine on cardiomyocyte apoptosis in mice infected with Coxsackie virus B3. *Chinese Journal of Nosocomiology*.

[87] Qian Z.-X., Huang H., Lin X.-J. (2009). Protective effects of tetramethylpyrazine on rat myocardial cells infected by Coxsackie virus B3 and its signal transduction mechanism. *Chinese Journal of Contemporary Pediatrics*.

[88] Ni S. H. (2005). Clinical observation of Shenmai and Ligustrazine injection in the treatment of children with acute viral myocarditis. *Journal of Emergency of Traditional Chinese Medicine*.

[89] Ma Z. L. (2011). To observe the clinical effect of Levocarnitine combined with Ligustrazine injection in the treatment of children with viral myocarditis. *China Medical Engineering*.

